# Identification of symptom clusters and sentinel symptoms during the first cycle of chemotherapy in patients with lung cancer

**DOI:** 10.1007/s00520-024-08600-5

**Published:** 2024-05-27

**Authors:** Yuanyuan Luo, Dongmei Mao, Le Zhang, Zhihui Yang, Jingxia Miao, Lili Zhang

**Affiliations:** 1 School of Nursing, Southern Medical University, Guangzhou, Guangdong, 510515 China; 2 Department of Medical Oncology, Nanfang Hospital, Southern Medical University, Guangzhou, Guangdong, 510515 China

**Keywords:** Symptom cluster, Sentinel symptom, Lung cancer, Walktrap algorithm, Apriori algorithm

## Abstract

**Purpose:**

To identify symptom clusters (SCs) in patients with lung cancer who are undergoing initial chemotherapy and to identify the sentinel symptoms of each SC.

**Methods:**

A convenience sampling method was used to recruit patients with lung cancer who were undergoing their initial chemotherapy treatment. Patient information was collected using the General Demographic Questionnaire, MD Anderson Symptom Inventory (including the lung cancer module) and a schedule documenting the initial occurrence of symptoms. The Walktrap algorithm was employed to identify SCs, while sentinel symptoms within each SC were identified using the Apriori algorithm in conjunction with the initial occurrence time of symptoms.

**Results:**

A total of 169 patients with lung cancer participated in this study, and four SCs were identified: the psychological SC (difficulty remembering, sadness, dry mouth, numbness or tingling, and distress), somatic SC (pain, fatigue, sleep disturbance, and drowsiness), respiratory SC (coughing, expectoration, chest tightness, and shortness of breath), and digestive SC (nausea, poor appetite, constipation, vomiting, and weight loss). Sadness, fatigue, and coughing were identified as sentinel symptoms of the psychological, somatic, and respiratory SCs, respectively. However, no sentinel symptom was identified for the digestive SC.

**Conclusion:**

Patients with lung cancer who are undergoing chemotherapy encounter a spectrum of symptoms, often presenting as SCs. The sentinel symptom of each SC emerges earlier than the other symptoms and is characterized by its sensitivity, significance, and driving force. It serves as a vital indicator of the SC and assumes a sentry role. Targeting sentinel symptoms might be a promising strategy for determining the optimal timing of interventions and for mitigating or decelerating the progression of the other symptoms within the SC.

## Introduction

Lung cancer is an increasingly serious global public health concern due to its substantial incidence and its role as a leading cause of cancer-related death [1]. In 2020, there were 2,206,771 new cases of lung cancer and 1,796,144 related deaths, and these figures are expected to increase [2, 3]. The methods for diagnosing and treating lung cancer have improved in recent years, thereby increasing the survival rate and extending the overall lifetime of patients [4]. Chemotherapy continues to be a cornerstone in the treatment of lung cancer, serving as an integral component of bi- or multimodality therapy, an adjunctive treatment following lung cancer resection and a palliative care option for patients with advanced-stage disease [5]. Despite its clinical success, chemotherapy is associated with many side effects. Due to the broad-spectrum toxic effects of chemotherapy, it may also affect normal cells while killing cancer cells, thus leading to chemotherapy-related toxicity [6, 7]. Patients with lung cancer endure a more significant symptom burden than those with other types of cancer. Symptoms such as pain, fatigue, and insomnia can substantially impact a patient’s quality of life [8, 9]. In particular, these symptoms frequently cooccur, forming a symptom cluster (SC) that intensifies the overall symptom burden [10]. Miaskowski and colleagues [11] defined an SC as two or more stable cooccurring symptoms that are independent of other clusters and may share underlying mechanisms. The occurrence of SCs can impede treatment processes, lead to depression and suicidal ideation among patients, and may even pose life-threatening risks [12, 13]. In addition, SCs not only inflict direct harm on lung cancer patients but also increase the caregiving burden for their family caregivers and the financial strain on the entire family [14, 15]. However, most current interventions targeting SCs are implemented after the symptoms manifest. Although the burden of symptoms can be alleviated, patients have already experienced the associated pain and incurred the related healthcare expenses due to delayed interventions [16–18]. Furthermore, healthcare providers have to deliver additional interventions to address the multiple symptoms that present in SCs.

The sentinel symptom is defined as a concurrent indicator or marker signifying the existence of an SC, which can provide a new perspective on symptom management [19]. Sentinel symptoms appear initially and exert an influence on the other symptoms within the SC [20]. If healthcare providers can identify sentinel symptoms and implement strategic interventions targeting SCs, they may be more effective in proactively advancing the timing of interventions and intervening in the subsequent progression of symptoms [21, 22]. However, the current research on sentinel symptoms remains insufficient. While a limited number of studies have identified sentinel symptoms, they often overlook the chronological order of symptom appearance or rely on a single method to identify SCs, thus neglecting the relationships between symptoms [19, 21, 23]. Consequently, a consensus on sentinel symptoms among lung cancer patients undergoing chemotherapy has yet to be established. The prevailing methods for identifying SCs include exploratory factor analysis (EFA) and principal component analysis (PCA). The SCs identified through various recognition methods also differ. However, previous studies have mostly relied on a single method for the identification of SCs and lack multiple methods for reconfirmation. Network analysis is a new approach to identifying SCs based on machine learning that can visually depict the relationships between symptoms using a network graph [24]. The Walktrap algorithm is a data-driven clustering algorithm that employs a sequence of random walks on a graph to identify clusters of symptoms within the network [25, 26]. It is crucial to understand the connections among symptoms and identify SCs using the Walktrap algorithm. However, few studies have employed this method to examine lung cancer patients undergoing chemotherapy. The Apriori algorithm is the most widely used algorithm for association analysis in data mining, and it can be used to explore potential relationships between symptoms [23, 27]. Research on sentinel symptoms is a growing field, and there is an urgent need to identify sentinel symptoms to effectively alleviate symptom burdens. Therefore, this study utilized the Walktrap algorithm to identify SCs and employed EFA to validate these clusters. Additionally, the Apriori method was combined with temporal sequencing to identify sentinel symptoms for each SC.

## Methods

### Patients and setting

A convenience sampling method was used. A total of 169 patients with lung cancer were recruited from May to November 2023 at Nanfang Hospital of Southern Medical University. The inclusion criteria were as follows: (1) first pathological diagnosis of lung cancer, (2) age 18 years or older, (3) initial chemotherapy treatment, and (4) provided informed consent and voluntarily participated in the study. The exclusion criteria were as follows: (1) major organic disease other than lung cancer, (2) severe cognitive impairment or mental illness, or (3) unable to communicate or write.

### Procedures

When patients initiated their initial chemotherapy cycle, the investigator explained the purpose and methods of the study. If patients agreed to participate, they were asked to complete questionnaires to assess the severity and timing of symptoms during their hospitalization. The researchers also informed patients that they had the option to withdraw from the study at any time without affecting their subsequent treatment and care. The participants were assured that all personal information would remain confidential and would only be used for research purposes. These questionnaires were collected at discharge.

### Instruments

#### Sociodemographic and clinical characteristics

The sociodemographic and clinical characteristics of the patients, including age, sex, body mass index (BMI), cancer stage, place of residence, educational level, monthly income, and financial burden, were recorded.

#### The Chinese version of the MDASI-C

The MD Anderson Symptom Inventory (MDASI-C) is a widely used patient-reported outcome measurement for assessing symptom burden. The MDASI was originally developed in 2000 by Cleeland CS at the University of Texas M. D. Anderson Cancer Center and subsequently translated into Chinese by Wang XS in 2004 [28, 29]. The MDASI-C comprises 19 items across two sections. The first section includes 13 items to assess the severity of core symptoms, including pain, fatigue, nausea, sleep disturbance, distress, shortness of breath, difficulty remembering, poor appetite, drowsiness, dry mouth, sadness, vomiting, and numbness or tingling. Each item is scored from 0 to 10, with 0 indicating not present and 10 representing as bad as you can imagine. The higher the score is, the more severe the symptoms are. The second section includes 6 items to assess the degree to which symptoms interfere with general activity levels, mood states, functioning at work, social interactions, walking ability, and overall enjoyment of life. Each item is scored from 0 to 10, with 0 indicating not interference and 10 indicating complete interference. The higher the score is, the stronger the impact the symptoms have on patients. The Cronbach’s *α* coefficient of the MDASI-C in this study was 0.883.

#### The Chinese version of the lung cancer module of the MDASI-LC

The Lung Cancer Module of the MD Anderson Symptom Inventory (MDASI-LC) is a module of the MDASI that was specifically developed for patients with lung cancer [30]. It contains three items: cough, constipation, and sore throat. Our research team previously translated and revised the MDASI-L to develop a modified lung cancer module suitable for Chinese patients (MDASI-LC) [31]. The MDASI-LC comprises six items—coughing, expectoration, hemoptysis, chest tightness, constipation, and weight loss—and has been verified to have good reliability and validity [32, 33]. The scoring criteria were the same as those for the MDASI, and the Cronbach’s *α* coefficient of the MDASI-LC in this study was 0.743. We appended a column to the end of each symptom entry to document the appearance time of symptoms.

### Data analysis

The data were analyzed using R 4.2.3 and SPSS Modeler 18.0. Normally distributed quantitative data, including age, BMI, and initial occurrence time of symptoms, are presented as the *mean* ± *standard deviation* (*x̅* ± s). Non-normally distributed data (i.e., the severity of symptoms) are expressed as the median and interquartile range (M (P25, P75)). Categorical data, including place of residence, educational level, work status, monthly income, financial burden, cancer stage, and the prevalence of 19 symptoms, are expressed as numbers and percentages (*n* (%)).

SCs were identified and visualized using the Walktrap algorithm, which was developed by Pons and Latapy and aims to detect connected components within a graph [34]. The Walktrap algorithm is preferred for the selection of community structures since it returns a dendrogram [35]. This algorithm identifies communities of nodes that exhibit relatively high interconnectivity, where nodes within a community are more prone to connect with other nodes in the same community [36]. In this study, the nodes represented symptoms and the communities represented SCs, which were visualized using distinct colors. To achieve an optimal configuration, the Fruchterman-Reingold algorithm was used to position nodes with stronger connections close to one another. In the EFA, symptoms with a frequency lower than 20% were excluded to enhance the clinical significance and ensure sufficient variability for conducting EFA. We employed principal component analysis and maximum variance (orthogonal) rotation to identify factors with eigenvalues exceeding 1 and symptom loadings exceeding 0.5. Statistical significance was defined as *P* < 0.05.

After identifying the SCs, we aimed to identified the sentinel symptom within each SC by using the Apriori algorithm and considering the initial occurrence time of symptoms. The Apriori algorithm is the most widely used algorithm for association analysis in data mining, as it enables the exploration of essential rules and potential connections among variables. Its effectiveness in identifying sentinel symptoms within SCs has been demonstrated [20, 23, 27]. Support and confidence are standard metrics in the Apriori algorithm for quantifying the association between symptoms. Support indicates the proportion of occurrences of both antecedent and consequent symptoms in all samples, while confidence indicates the proportion of cases where the consequent symptom appears after the antecedent symptom. This aligns with the concept of sentinel symptoms: sentinel symptoms that occur initially and subsequently influence others. In this study, a valid relationship between two symptoms was identified if the preceding item had a support value greater than 40%, a confidence value greater than 60%, and if the confidence value was higher than the support value.

## Results

### Sociodemographic and clinical characteristics

A total of 169 patients were included in this study, with an average age of 57.82 ± 10.61 years and an average BMI of 22.31 ± 3.22. The majority of the participants were male (104 (61.54%)). Furthermore, a significant proportion of patients had advanced-stage cancer (128 (75.74%)]. The sociodemographic and clinical characteristics of the participants are shown in Table 1.

**Table 1:** Sociodemographic and clinical characteristics of the participants (*n* = 169)

Patient characteristics	*n* (%)
Age (years)	
*M* ± *SD*	57.82 ± 10.61
BMI	
*M* ± *SD*	22.31 ± 3.22
Sex	
Male	104 (61.54)
Female	65 (38.46)
Place of residence	
Urban	71 (42.01)
County or Town	34 (20.12)
Rural	64 (37.87)
Educational level	
Junior high school and below	103 (60.95)
Senior high school	39 (23.08)
Graduate and above	27 (15.98)
Work status	
Working or self-employed	90 (53.25)
Unemployed	34 (20.12)
Retirement	45 (26.63)
Monthly income (China yuan)	
< 3000	34 (20.12)
3000–6000	73 (43.19)
≥ 6000	62 (36.69)
Financial burden	
Not at all	10 (5.92)
A little bit	87 (51.48)
Quite a bit	57 (33.73)
Very much	15 (8.87)
Cancer stage	
Stage I	11 (6.51)
Stage II	30 (17.75)
Stage III	42 (24.85)
Stage IV	86 (50.89)

### Prevalence, severity, and initial occurrence time of symptoms

During the first cycle of chemotherapy, the most common symptoms experienced by lung cancer patients were sleep disturbance [156 (92.31%)] and fatigue [154 (91.12%)], while the least prevalent symptom was hemoptysis [22 (13.02%)]. The most severe symptoms included fatigue, sleep disturbance, and pain. In addition, coughing, expectoration, and sadness were the earliest symptoms to appear, while constipation was the latest symptom (Table 2).

**Table 2:** Prevalence and severity of 19 symptoms

No	Symptom	Prevalence (*n* (%))	Severity (*M* (P25, P75))	Initial occurrence time of symptoms [hours, (*M* ± *SD*)]
1	Pain	118 (69.82)	2.0 (0.0, 4.0)	31.37 ± 5.13
2	Fatigue	154 (91.12)	4.0 (2.0, 6.5)	26.53 ± 7.46
3	Nausea	59 (34.91)	0.0 (0.0, 1.0)	28.13 ± 10.01
4	Sleep disturbance	156 (92.31)	3.0 (2.0, 5.0)	31.21 ± 8.50
5	Distress	138 (81.66)	1.0 (1.0, 2.0)	40.01 ± 8.49
6	Shortness of breath	94 (55.62)	1.0 (0.0, 2.0)	30.94 ± 10.88
7	Difficulty remembering	102 (60.36)	1.0 (0.0, 3.0)	50.51 ± 12.00
8	Poor appetite	104 (61.54)	1.0 (0.0, 3.5)	22.06 ± 3.52
9	Drowsiness	93 (55.03)	1.0 (0.0, 3.0)	35.62 ± 11.21
10	Dry mouth	106 (62.72)	1.0 (0.0, 3.0)	28.32 ± 9.04
11	Sadness	103 (60.95)	1.0 (0.0, 2.0)	19.58 ± 6.40
12	Vomiting	37 (21.89)	0.0 (0.0, 0.0)	38.87 ± 11.62
13	Numbness or tingling	89 (52.66)	1.0 (0.0, 2.0)	37.00 ± 10.16
14	Coughing	103 (60.95)	1.0 (0.0, 3.0)	13.56 ± 6.93
15	Expectoration	80 (47.34)	0.0 (0.0, 2.0)	13.94 ± 6.26
16	Hemoptysis	22 (13.02)	0.0 (0.0, 0.0)	59.30 ± 15.50
17	Chest tightness	75 (44.38)	0.0 (0.0, 2.0)	34.17 ± 8.78
18	Constipation	39 (23.08)	0.0 (0.0, 0.0)	87.15 ± 21.51
19	Weight loss	80 (47.34)	0.0 (0.0, 1.0)	30.19 ± 7.63

### Symptom clusters of lung cancer patients undergoing initial chemotherapy

**Fig. 1:**
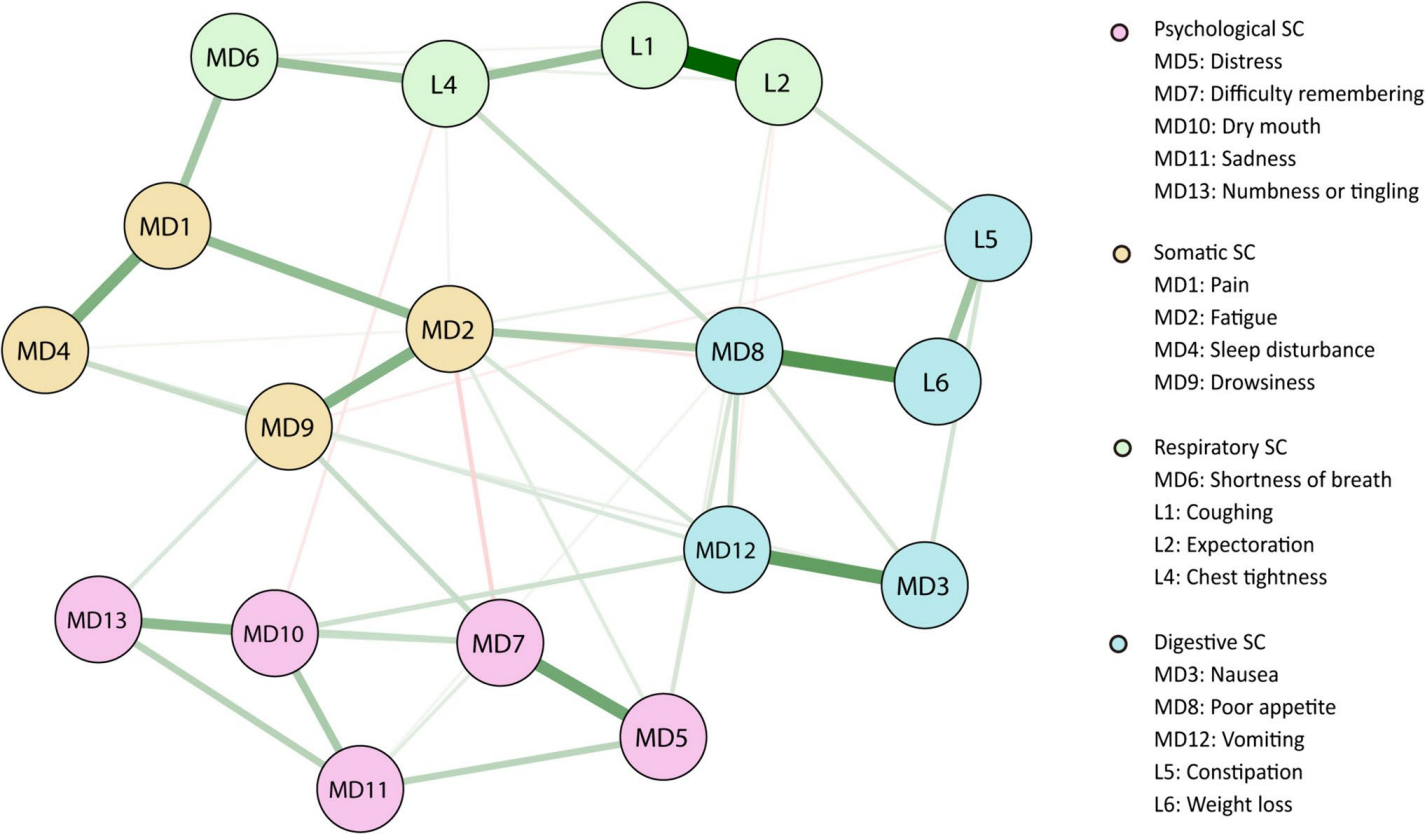
Identification and visualization of symptom clusters using the Walktrap algorithm

The Walktrap algorithm, a reliable method for community detection, was used to identify four SCs in this study. These results are visually depicted in a network diagram (Fig. 1). Each ball represents a symptom, and the thickness of the edge indicates the strength of the correlation. The thicker the edge is, the stronger the correlation, and vice versa. SCs and their respective nodes are depicted in different colors. The first SC included distress, difficulty remembering, sadness, numbness or tingling, and dry mouth. It was referred to as the psychological SC (pink ball) according to the characteristic composition of symptoms. The second SC was the somatic SC (yellow ball), which included pain, fatigue, sleep disturbance, and drowsiness. The third SC was the respiratory SC (green ball), which included coughing, expectoration, chest tightness, and shortness of breath. The last SC consisted of gastrointestinal symptoms and was therefore called the digestive SC (blue ball), which included nausea, poor appetite, constipation, vomiting, and weight loss.

We used EFA to confirm the constituent components of the SCs. Hemoptysis, which was experienced by less than 20% (13.02%) of patients, was excluded from the analysis; the remaining symptoms were included in the EFA. The EFA identified four SCs that accounted for a cumulative variance of 60.02%. This result is consistent with the Walktrap algorithm and validates the accuracy of identifying these four SCs. The Kaiser–Meyer–Olkin (KMO) coefficient was 0.791, and the *P* value of Bartlett’s sphericity was less than 0.001. The internal consistency coefficients of the four SCs were 0.772, 0.765, 0.784, and 0.770, respectively (Table 3).

**Table 3:** Symptom clusters identified by EFA (*n* = 169)

Symptom cluster	Symptom	Factor loading			
		Factor 1	Factor 2	Factor 3	Factor 4
Psychological symptom cluster	Difficulty remembering	**0.777**	-0.002	0.102	0.110
	Sadness	**0.731**	0.130	0.003	0.145
	Dry mouth	**0.685**	0.164	-0.107	0.125
	Numbness or tingling	**0.726**	0.070	-0.072	0.004
	Distress	**0.577**	0.100	0.225	0.181
Somatic symptom cluster	Pain	0.039	**0.786**	0.180	-0.008
	Fatigue	0.067	**0.729**	0.094	0.283
	Sleep disturbance	0.113	**0.703**	0.074	0.082
	Drowsiness	0.384	**0.631**	0.025	0.120
Respiratory symptom cluster	Coughing	0.039	0.087	**0.856**	0.134
	Expectoration	0.198	-0.067	**0.849**	0.100
	Chest tightness	-0.167	0.246	**0.652**	0.181
	Shortness of breath	0.004	0.433	**0.560**	0.027
Digestive symptom cluster	Weight loss	0.202	0.012	0.200	**0.742**
	Nausea	0.086	0.382	-0.026	**0.709**
	Poor appetite	0.300	0.265	0.257	**0.684**
	Constipation	-0.009	-0.154	0.352	**0.645**
	Vomiting	0.238	0.404	-0.249	**0.593**
Cronbach’s α		0.772	0.765	0.784	0.770

### Sentinel symptom of each symptom cluster

In the psychological SC, sadness was the first symptom to appear. According to the Apriori algorithm and chronological order, when sadness serves as an antecedent symptom and other symptoms are considered consequences, the support value is greater than 40%, the confidence value is greater than 60%, and the confidence value is greater than the support value. Therefore, sadness can be identified as the sentinel symptom of the psychological SC. Based on the three principles of the Apriori algorithm and the order of occurrence, fatigue was identified to be the sentinel symptom of the somatic SC, and coughing was identified to be the sentinel symptom of the respiratory SC (Table 4). In addition, the results of the Apriori algorithm for the digestive SC were consistent with the aforementioned principles; however, weight loss was not the initial symptom for this SC, thus suggesting that there was no sentinel symptom for the digestive SC.

**Table 4:** Apriori algorithm-based association rules

Symptom cluster	Antecedent symptom	Consequent symptoms	Support (%)	Confidence (%)
Psychological symptom cluster	Dry mouth	Distress	62.72	82.08
		Sadness	62.72	66.04
		Difficulty remembering	62.72	64.15
	Difficulty remembering	Distress	60.36	83.33
		Dry mouth	60.36	66.67
		Sadness	60.36	65.69
	**Sadness**	Distress	60.95	93.20
		Dry mouth	60.95	67.96
		Difficulty remembering	60.95	65.05
	Numbness or tingling	Distress	52.66	83.15
		Dry mouth	52.66	71.91
		Sadness	52.66	64.05
		Difficulty remembering	52.66	62.92
Somatic symptom cluster	**Fatigue**	Sleep disturbance	91.12	93.51
	Pain	Fatigue	69.82	95.76
		Sleep disturbance	69.82	94.92
	Drowsiness	Sleep disturbance	55.03	95.70
		Fatigue	55.03	94.62
		Pain	55.03	75.27
Respiratory symptom cluster	**Coughing**	Expectoration	60.95	73.79
		Shortness of breath	60.95	67.96
	Shortness of breath	Coughing	55.62	74.47
	Expectoration	Coughing	47.34	95.00
		Shortness of breath	47.34	68.75
		Chest tightness	47.34	62.50
	Chest tightness	Coughing	44.38	81.33
		Shortness of breath	44.38	70.67
		Expectoration	44.38	66.67
Digestive symptom cluster	Weight loss	Poor appetite	47.34	65.00

## Discussion

In this study, SCs were identified using the Walktrap algorithm and were further validated by EFA. Both methods yielded the same four SCs, namely, the psychological SC, somatic SC, respiratory SC, and digestive SC. The results are consistent with the findings reported by Wong et al. [37], thereby validating the feasibility of employing the Walktrap algorithm for identifying and visualizing SCs in lung cancer patients. Furthermore, we utilized a combination of the Apriori method and the initial occurrence time of symptoms to identify sentinel symptoms. This valuable information enables healthcare providers to target specific interventions for symptom management, thereby alleviating symptom-induced suffering and optimizing health care.

The psychological SC included sadness, distress, difficulty remembering, numbness or tingling, and dry mouth. Patients with lung cancer frequently experience emotional distress, which can adversely affect their physical and mental well-being [38]. The diagnosis of cancer itself constitutes a significant stressor for patients, giving rise to emotional responses such as sadness and distress. Chemotherapy drugs may induce uncomfortable symptoms, including dry mouth, numbness, and tingling. Sometimes, cognitive impairment or difficulty remembering may occur due to brain function impairments, further contributing to the psychological burden [39]. In addition, the sentinel symptom of the psychological SC was sadness. Due to the uncertainties surrounding cancer and its treatment, patients often engage in contemplation about their future and its potential impact on their family, thus triggering an initial sense of sadness. These emotional reactions can impact hormone levels and immune system functioning, resulting in reduced saliva production and heightened perceptions of numbness and pain [40, 41]. Therefore, it is imperative for healthcare providers to promptly recognize the sadness of patients, provide them with adequate emotional support, and collaborate with their families to effectively assist them in navigating both the emotional and physical challenges they may face. In addition, encouragement from fellow patients is crucial. Inviting patients who have experienced positive treatment outcomes can effectively reduce negative emotions among patients, thereby alleviating the symptom burden.

Pain, fatigue, sleep disturbance, and drowsiness constituted the somatic SC, and fatigue was the sentinel symptom of this SC, which was consistent with the findings of Ma and Ju [23, 27]. Fatigue, pain, and sleep disturbances are interconnected physiological mechanisms that mutually influence each other [42]. Tumors and treatment can stimulate an inflammatory response, which activates the immune system and triggers the release of inflammatory factors and white blood cells. This disturbance to normal energy metabolism contributes to fatigue. Inflammatory factors extend their impact on the endocrine system, resulting in dysfunction of the hypothalamic‒pituitary‒adrenal axis and thus leading to sleep disorders [43]. Moreover, inflammation may disrupt the balance of neurotransmitters, thereby affecting the nervous system and increasing the perception of pain [44]. Fatigue induces a reduction in daytime activity, disturbing the circadian rhythm and influencing sleep patterns [45]. Insufficient sleep, in turn, exacerbates daytime drowsiness caused by fatigue and intensifies the perception of pain [46]. This establishes a detrimental cycle that amplifies the symptom burden for patients. Therefore, intervening in fatigue becomes crucial for interrupting this cycle, thus slowing or alleviating the tendency for these symptoms to exacerbate each other. In clinical practice, healthcare providers could encourage lung cancer patients to engage in regular physical activity based on their individual conditions. Activities such as walking, swimming, aerobics, or resistance exercises are beneficial. Additionally, adhering to an anti-inflammatory diet can help reduce the occurrence of fatigue.

Coughing, expectoration, chest tightness, and shortness of breath collectively constitute the respiratory SC. Lung tumors or pleural effusion can result in airway obstruction, contributing to manifestations such as coughing, chest tightness, and shortness of breath [47]. Additionally, chemotherapy medications can induce systemic inflammation, resulting in congestion of the respiratory mucosa and increased production of secretions [48]. Consequently, this can manifest as symptoms such as coughing and expectoration. Furthermore, these drugs may stimulate bronchoconstriction and restrict airflow, leading to breathing difficulties and a sensation of chest tightness. The sentinel symptom of the respiratory SC was coughing. When mucus accumulates in the airway, patients typically experience coughing as the initial symptom, followed by expectoration. Coughing leads to changes in airflow and lung capacity within the chest, resulting in chest tightness. The irritation caused by coughing can also provoke spasms in the airways, which restricts airflow and causes breathlessness. Consequently, patients should be encouraged to increase their water intake and cease smoking to alleviate coughing. Moreover, the incorporation of respiratory training and the practice of effective coughing techniques can further contribute to symptom reduction.

The digestive SC included nausea, poor appetite, constipation, vomiting, and weight loss. The functioning of the digestive system can be influenced by neuromodulation, immune system factors, and tumor-related factors [49]. Patients who experience significant mental stress or feelings of melancholy may also experience disturbances in their appetite and digestive function. The presence of tumors and their treatment can trigger an inflammatory response in the body, leading to the release of cytokines that can further impact normal gastrointestinal functioning, resulting in issues such as indigestion, poor appetite, and nausea [50]. Additionally, the use of pain medications has been identified as a potential contributor to constipation [51]. However, owing to variations in individuals’ stress tolerance and responses to treatment, no consistent pattern can be observed in the sequence of symptoms.

## Limitations

The present study has several limitations that need to be considered. First, we employed convenience sampling to select patients from a single hospital, potentially limiting the generalizability of our findings to the broader population. Therefore, it is highly recommended to conduct a multicenter study to validate the findings of this study. Additionally, the determination of symptom occurrence relied solely on subjective patient reports. In future research endeavors, incorporating objective biomarkers alongside patient-reported symptoms could enhance the precision and comprehensiveness of symptom identification.

## Conclusion

The present study utilized the Walktrap algorithm to identify SCs and employed EFA to validate these clusters among lung cancer patients. Moreover, the identification of the sentinel symptom for each cluster was achieved by integrating the initial occurrence time of symptoms and the Apriori algorithm. These findings can assist healthcare providers in developing targeted strategies for managing symptoms, enabling earlier intervention, reducing patient burden, and optimizing medical resources.

## Data Availability

The data supporting the findings of this study are available from the corresponding author upon reasonable request.
